# Agricultural wastes from wheat, barley, flax and grape for the efficient removal of Cd from contaminated water

**DOI:** 10.1039/c8ra07877g

**Published:** 2018-12-04

**Authors:** Patrick M. Melia, Rosa Busquets, Santanu Ray, Andrew B. Cundy

**Affiliations:** Kingston University, Faculty of Science, Engineering and Computing Kingston Upon Thames KT1 2EE UK patrick-melia@outlook.com r.busquets@kingston.ac.uk; Surface Analysis Laboratory, University of Brighton, Faculty of Science and Engineering BN2 4GJ UK; University of Southampton, School of Ocean and Earth Science Southampton SO14 3ZH UK

## Abstract

Agricultural production results in wastes that can be re-used to improve the quality of the environment. This work has investigated for the first time the use of abundant, un-modified agricultural wastes and by-products (AWBs) from grape, wheat, barley and flax production, to reduce the concentration of Cd, a highly toxic and mobile heavy metal, in contaminated water. At concentrations of 1.1 mg Cd per L, flax and grape waste were found superior in removing Cd compared with a granular activated carbon used in water treatment, which is both more expensive and entails greater CO_2_ emissions in its production. At a pH representative of mine effluents, where Cd presents its greatest mobility and risk as a pollutant, grape and flax waste showed capacity for effective bulk water treatment due to rapid removal kinetics and moderate adsorption properties: reaching equilibrium within 183 and 8 min – adsorption capacities were determined as 3.99 and 3.36 mg Cd per g, respectively. The capacity to clean contaminated effluents was not correlated with the surface area of the biosorbents. Surface chemistry analysis indicated that Cd removal is associated with exchange with Ca, and chemisorption involving CdCO_3_, CdS and CdO groups. This work indicates that some AWBs can be directly (*i.e.* without pre-treatment or modification) used in bulk to remediate effluents contaminated with heavy metals, without requiring further cost or energy input, making them potentially suitable for low-cost treatment of persistent (*e.g. via* mine drainage) or acute (*e.g.* spillages) discharges in rural and other areas.

## Abstract 2

Agricultural production results in wastes that can be re-used to improve the quality of the environment.

## Graphical abstract

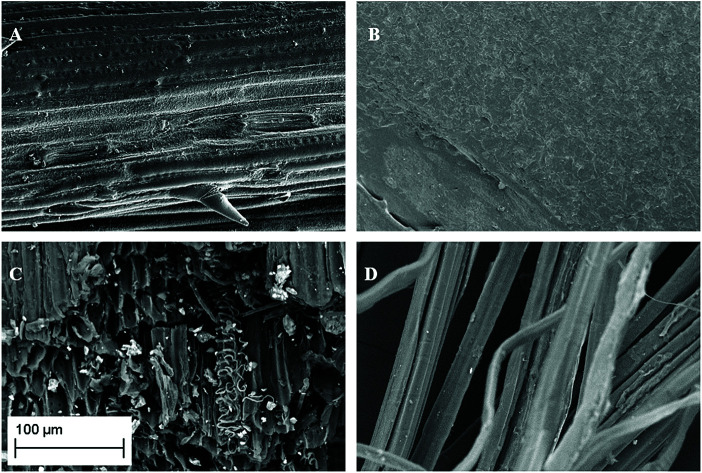

## 1 Introduction

Heavy metals, when present in the environment in high concentrations, can cause detrimental effects in a variety of organisms including humans. Several heavy metals are micronutrients while others are potent cell toxins but almost all are harmful if critical thresholds for toxicity are exceeded.^1^ Surface water is one of the first environmental compartments at risk of pollution by heavy metals as it may receive a range of industrial and domestic effluents, as well as urban run-off and landfill leachate.^2,3^

Among heavy metals, Cd has special relevance due to its broad use, mobility and toxicity (classified as carcinogenic, mutagenic, toxic for reproduction and toxic to bones and kidneys).^4,5^ Furthermore, Cd ions are considered to be more mobile in aquatic environments than most other heavy metals.^4^ The global extraction of Cd was 20 100 tons in 2009 ^6^ and a fraction of this ends up being released to the environment, and Cd is found at toxic concentrations in some rivers.^2^ Indirect sources of Cd to surface water can be emissions or products from industries such as electroplating, pigment/textile, plastics production (*i.e.* with up to 2 g Cd per kg PVC), Ni–Cd batteries, and photovoltaic technologies (in the form of Cd–tellurium).^7^ Mineral fertilisers are derived from phosphate rock where Cd occurs naturally at concentrations of 1–92 mg Cd per kg rock,^8,9^ consequently Cd can also enter directly to surface water, soil and the food chain through direct application of phosphatic fertilisers, which represents 60% of the emissions of Cd to soils.^10,11^ Among the heavy metals in phosphate rock, Cd has the highest transfer factor in plants,^8^ and therefore its concentration in water and soil is of high concern. Cd can also be present in mine effluents which, in origin, may be very acidic (pH 2.2–3.1),^12^ conditions in which Cd presents its highest mobility. The pH of these effluents rises later to 4–5 when mixing with pristine waters.^12^

Cd is identified as a priority hazardous substance, listed among the 33 substances identified as such by the European Commission.^13^ The discharge of Cd from various industrial sectors into surface water is to be ceased by 2020,^14^ and its limit in drinking water is 5 μg L^−1^.^15^ The removal of Cd from industrial effluents, mine waters or other Cd sources is therefore of high importance to prevent its discharge and accumulation in the environment. A recent review has discussed the current technologies used for the removal of Cd and other heavy metals from industrial wastewater.^16^ Chemical precipitation is the most widely used technique in industry for Cd removal, and ion exchange techniques are also commonly used. Other techniques include membrane filtration, coagulation and flocculation, flotation, electrochemical treatment and adsorption *via* activated carbon. However, despite being highly effective, there are practical drawbacks that limit the applicability of these approaches such as the high volumes of chemical sludge generated in chemical precipitation; and the high cost (and potential for biofouling) of ion exchange and membrane filtration-based processes.^16^ Adsorption has become a popular treatment for the removal of Cd and is considered one of the most efficient and economical contaminant removal techniques.^17^ Biochar and activated carbons have been used for the adsorption of Cd, and although effective, they are relatively expensive to produce, which limits their use.^18^ Numerous low-cost materials have been investigated for their potential as alternatives to activated carbon, and these include natural materials such as zeolite^19^ and montmorillonite;^20^ or industrial wastes such as fly ash.^21^ There has also been recent interest in the application of agricultural wastes and by-products (AWBs) for the removal of Cd such as leaves from camphor tree^22^ or garlic peel.^23^ These sorbents typically have low preparation costs, and potentially greener credentials than activated carbons, *i.e.* they use natural agricultural residues rather than coal-derived products as a feedstock and can be applied to divert wastes from landfill or other disposal. Much of the published data however have focused on processed or modified AWBs, and on adsorption capacity and kinetics rather than fundamental adsorption mechanisms.

This study aims to evaluate abundant, un-modified agricultural wastes and by-products as cost-effective sorbents for the removal of Cd from water. Surface chemistry analysis is used to assess adsorption mechanisms for Cd, and adsorption kinetics and capacities compared to those previously reported for processes or modified AWBs. This research contributes to the environmentally sustainable initiative of the development of a more circular economy, where the utilisation of food wastes can be used to improve water quality (water-food nexus).

## 2 Materials and methods

### 2.1 Chemicals and materials

AWBs were selected to represent a range of bulk agricultural products and wastes from cereal, wine and oil production. Six AWBs: grape wastes (GW), flax wool (FW), flax mat (FM), flax shive (FS), barley straw (BS) and wheat straw (WS), all dried in air, were sourced directly from producers. The grape wastes were mainly skins, with some seeds and stalks attached, and were by-products from the production of Tempranillo (red) wine sourced from DO Penedès (NE Spain). The flax (Linum usitatissimum) materials, were obtained from the FP7 Interreg IVA (South) project 4044 “Flax – Increasing its value for society” through the collaboration of the University of Brighton with Linière de Bosc Nouvel S.A., Seine-Maritime (France). The wheat and barley straws were sourced from the John Innes Centre (UK) and NE Spain (Penedès), respectively. The sorbents were further dried in a vacuum oven at 40 °C before use. The AWBs were cut to achieve approximately equal particle sizes (1 cm) and their different natural widths were not modified. A commercial granular activated carbon (GAC), provided by Anglian Water (UK), was also used in the study as a commercially used sorbent for comparison. A stock solution 1000 mg L^−1^ Cd solution (in water, 2% HNO_3_) (PerkinElmer Pure, ISO certified) was further diluted to make up all solutions with deionized water. Where pH has been adjusted it was done so dropwise using 1 M NaOH and HNO_3_ solutions.

### 2.2 Surface chemistry characterisations

Surface area measurements (*S*_BET_) of the AWBs were obtained using N_2_ adsorption isotherms at 77 K, after degassing the airdried samples (2 g) for 24 h at 90 °C and treating adsorption/desorption data with BET (Brunauer–Emmett–Teller) and BJH (Barrett–Joyner–Halenda) modelling as described elsewhere using an Autosorb adsorption analyser (Quantachrome Instruments, USA).^24^

The samples dried in air were sputter-coated with palladium and examined using a JEOL JSM-6310 Field Emission Scanning Electron Microscope (Oxford instruments, UK) operating at 3–5 KV for all sorbents.

XPS was performed using an ESCALAB 250 Xi system (Thermo Scientific) equipped with a monochromated Al Kα X-ray source, a hemispherical electron energy analyzer, a magnetic lens and a video camera for viewing the analysis position. The standard analysis spot of *ca.* 900 × 600 μm^2^ was defined by the microfocused X-ray source. Full survey scans (step size 1 eV, pass energy 150 eV, no of scans: 5, dwell time 50 mS) and narrow scans (step size 0.1 eV, pass energy 20 eV, no of scans: 10, dwell time 100 mS) of the C 1s (binding energy, BE ∼ 285 eV), O 1s (BE ∼ 531 eV), P 2p (BE ∼ 130 eV), Cd 3d (BE ∼ 410 eV), Ca 2p (BE ∼ 350 eV), and Cu 2p (BE ∼ 940 eV) regions were acquired from three separate areas on each sample. Data were transmission function corrected and analyzed using Thermo Avantage Software (Version 5.952) using a smart background. The XPS analysis was carried out on dry grape waste before and after Cd adsorption studies.

### 2.3 Adsorption studies

Batch uptake studies aimed at screening the performance of the AWBs were carried out by mixing 40 mL of contaminated aqueous solutions representative of mine effluent (19.3 mg Cd per L at pH 2.2)^12,25^ and 0.2 g of each individual AWB in 50 mL polypropylene centrifuge tubes. These were shaken at 100 rpm at a temperature of 22 ± 2 °C in an orbital shaker for 48 h before separation by centrifugation. The mass of Cd sorbed per mass of AWB was calculated from the difference between the initial (*C*_i_) and final (*C*_eq_) concentration in solution. Sorption removal efficiency (%) and capacity, *q*_eq_ (mg g^−1^ of dry material), were calculated as follows:1
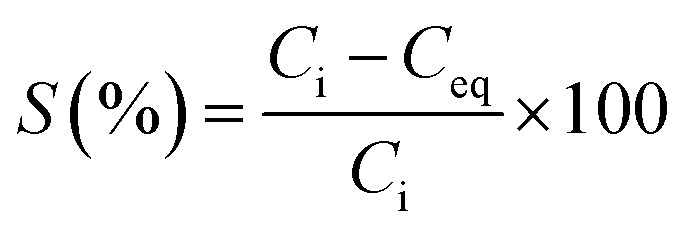
2
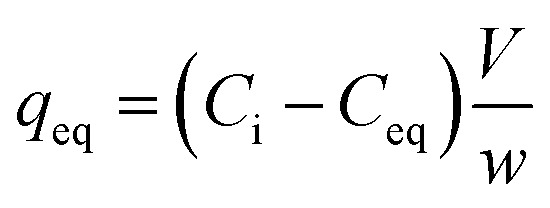
where *V* (L) is the volume of solution and *w* (g) is the amount of sorbent used. In all studies, control samples (solution without AWB added) were conducted under the same experimental conditions – the resulting Cd concentration in the control is used as *C*_i_ in eqn (1) and (2).

For investigation of sorption kinetics, uptake was measured over several predetermined time intervals (1, 5, 20, 60, 120, 240 and 480 minutes) in duplicate from an initial concentration of 18.4 mg Cd per L. The pH was adjusted to 5.5 as described in Section 2.1 – this has been indicated as the optimal acidity for Cd adsorption^26,27^ and is representative of acidic soils and mine effluents that have been attenuated.^12^ No effort was made to maintain pH throughout the process. The experimental data obtained was modelled using kinetic models (pseudo-first- and pseudo-second-order).

The effect of the concentration of contaminant on its removal was assessed using initial concentrations of ∼1.1, 6.8 and 21.5 mg Cd per L at pH 5.5, performed at equilibria with a contact time of 4 hours (determined through the kinetics experiments). A commercial granular activated carbon (AC) was introduced here to provide a comparison against the AWBs at low concentration. The quantification of Cd was carried out using an ICP-OES Perkin Elmer Optima™ 2100 DV with detection limit in the analysis of Cd at <10 ppb.

## 3 Results and discussion

Derived products from the production of grapes/wine, flax, barley, and wheat were selected because these crops have high global production rates, for example: 247 million hectolitres of wine produced in 2017;^28^ 309 000 tonnes of flax fibre in 2015;^29^ 136 million tonnes of barley in 2007;^30^ and 754 million tonnes of wheat production forecast for 2018/19.^31^

### 3.1 Structural characterisation of the AWBs

High surface area is an advantageous characteristic in sorbents, as the abundance of active sites involved in the uptake of contaminants is maximised. The six AWBs tested were characterised by N_2_ adsorption isotherms and the surface areas are presented in Table 1. *S*_BET_ values of the AWB materials were all <10 m^2^ g^−1^, excluding flax wool which had a surface area of 75.1 m^2^ g^−1^. Commercial GAC showed much higher surface area (552 m^2^ g^−1^). Hence, AWBs are all characterised by relatively low surface areas, which may decrease their value as an adsorbent; however, their low or zero cost economically favours their use in a large scale.

**Table 1:** *S*_BET_ values (m^2^ g^−1^) for the AWB materials studied and commercial granular activated carbon (GAC). Obtained by N_2_ adsorption at 77 K

Material	Surface area (m^2^ g^−1^)
Grape wastes	1.6
Flax wool	75.1
Flax shive	1.5
Flax mat	4.2
Wheat straw	8.7
Barley straw	9.3
Granular activated carbon (GAC)	552

**Fig. 1:**
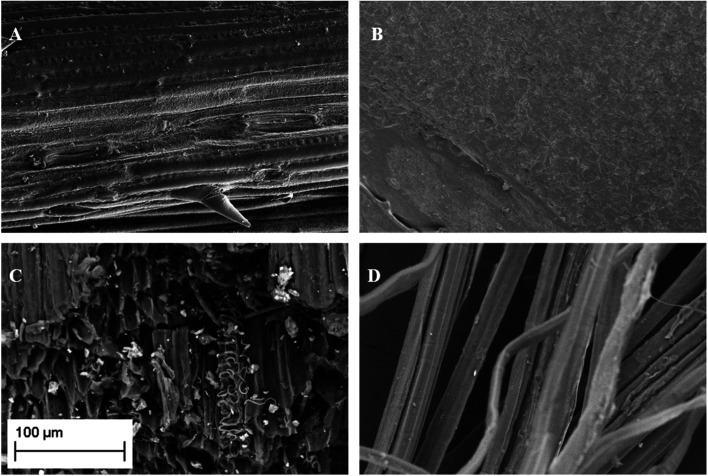
SEM micrographs of wheat straw (A), grape waste (B), flax shive (C) and flax wool (D). The scale bar given is applicable to all the micrographs.

SEM observations (Fig. 1) showed the existence of macroporosity in some samples. The morphology of the samples is distinct, from amorphous structures with little evidence for ordered pore structure (Fig. 1B) to fibrous structures (Fig. 1D) and samples with well-defined channels, still in the range of macropores (Fig. 1A and C). Such pore structures can act as transport routes for Cd-containing solutions to access inner pores and sorption sites. The existing porosity represents the native plant structure of the materials. The higher porosity and surface areas associated with GAC materials are derived through activation processes at high temperature, which requires the input of energy, CO_2_, and leads to increase of micro and mesoporosity, resulting in greater surface area.

### 3.2 Cd sorption

**Fig. 2:**
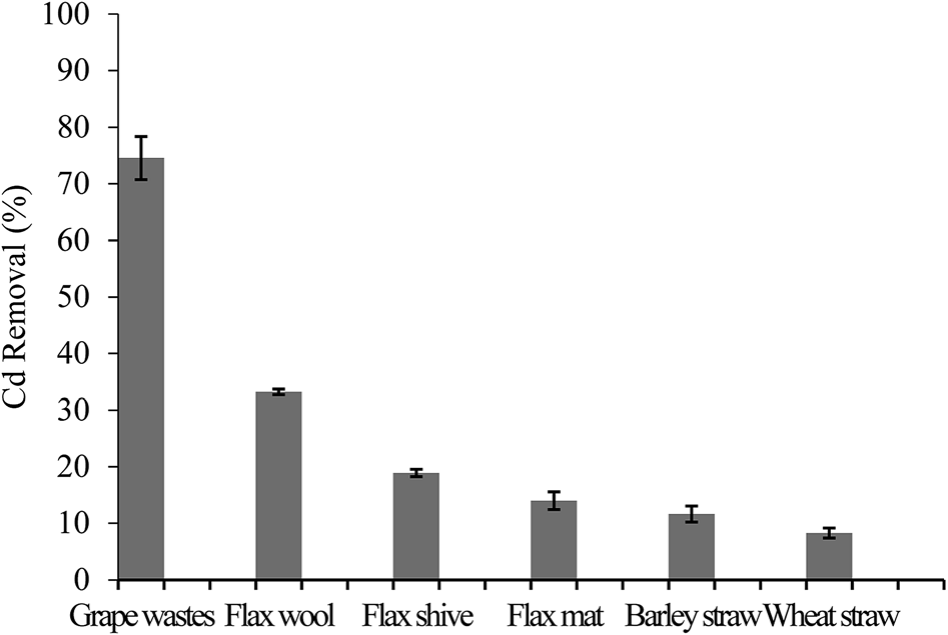
Removal efficiencies (%) of all tested AWB materials. Initial Cd concentration of 19.3 mg L^−1^ in batch conditions (200 mg sorbent: 40 mL of solution, 100 rpm 24 h, pH 2.2). Error bars represent the standard deviation (*n* = 3).

The capacity of the AWBs to uptake Cd was primarily determined in a screening study under conditions using Cd concentrations typical of those found in mine drainage,^32^ although this is at significantly greater concentrations of Cd than typically found in environmental waters. Use of such high concentrations however allows comparison of the different sorbents at or near to their maximum adsorption capacities. The removal efficiency of Cd by the AWBs is presented in Fig. 2.

The Cd sorption efficiency of grape waste was significantly higher than the other AWBs tested, giving a removal of 74.6 ± 4.3% Cd in this study, despite the very low surface area of this material (Table 1). In contrast, both types of straw showed lower potential for the removal of Cd from solution, with removal efficiencies of <12%. Of the three flax-based materials, flax wool performed best although this was still less than half as efficient as grape wastes. The results also indicate that the processing stages used when converting the wool into a flax mat can lead to physico-chemical changes in the flax sorbent that can reduce the uptake of Cd.

**Fig. 3:**
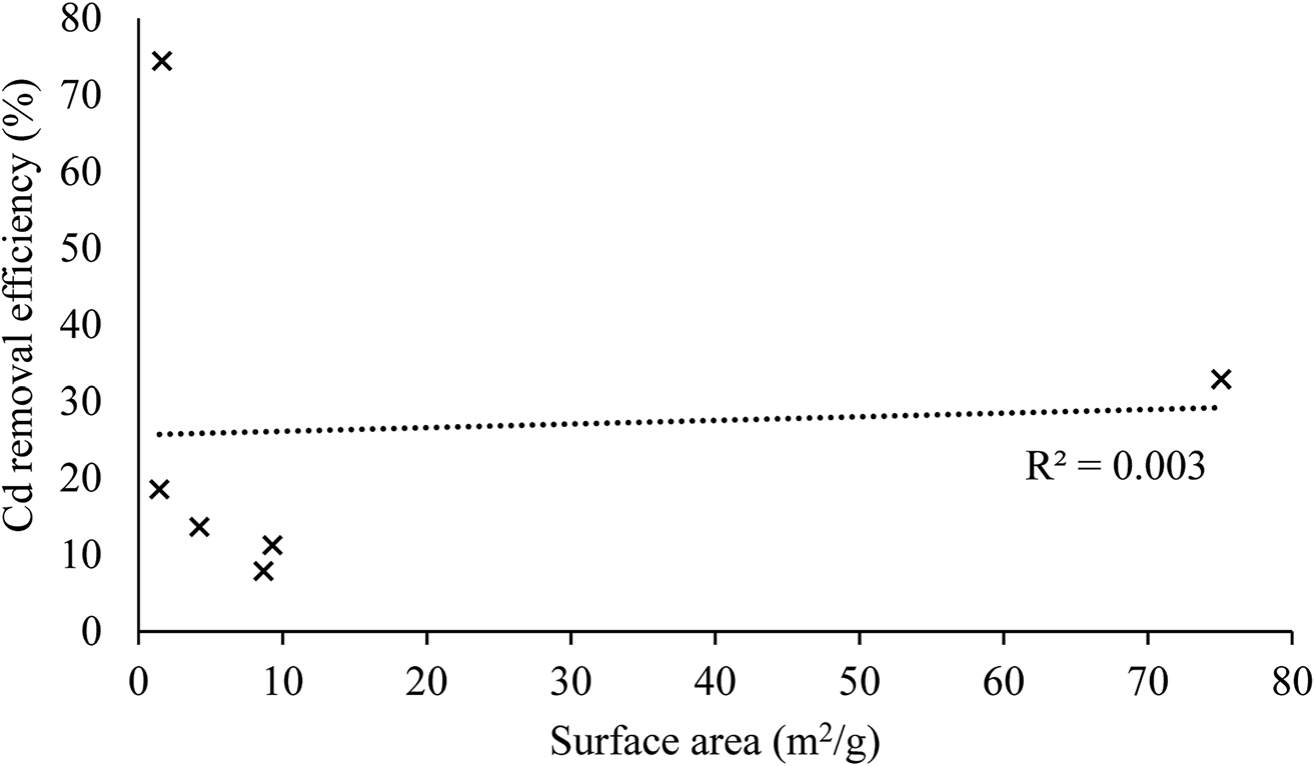
Plot of Cd removal efficiency (%) against surface area (*S*_BET_, m^2^ g^−1^) for the 6 AWBs tested.

The lack of correlation between surface area and Cd removal efficiency (Fig. 3) indicates that there is some degree of selectivity in the adsorption process. Earlier work found that the phenolic moiety from the lignan secoisolariciresinol diglucoside from flax seeds (not assayed in our work) could complex divalent cations.^33^ Grape wastes are also known to be rich in phenolic moieties.

Based on these preliminary results, grape and flax wool were chosen for further study. They represent the two most efficient waste materials studied (in terms of sorption properties) and incorporate varying features amongst the AWBs which may be important for further understanding mechanisms driving the adsorption of Cd to these biosorbents.

### 3.3 Effect of initial Cd concentration

Table 2 shows the efficiency of the sorbents to remove Cd from different initial concentrations at pH 5.5. Grape waste achieves >90% removal of Cd from initial concentrations ranging from 1.1–21.5 mg Cd per L; flax wool removes more than 90% of Cd from the lower initial concentration and 78.1% from the highest initial concentration tested. The removal of Cd is pH dependent as removal efficiency at ∼20 mg Cd per L is lower at pH 2.2 (Fig. 1) than at nearer neutral pH (Table 2) – this is in agreement with other studies using other biomass such as biochar.^34^ Activated carbon was also tested at the lowest initial concentration and was found to be less efficient than both agricultural materials tested (removing 34 ± 4.9% of Cd from solution). This indicates that AWBs and GAC have different adsorption mechanisms, with the AWBs having a higher heat of adsorption and offering better performance than GAC at this concentration, and at lower cost.

**Table 2:** Sorption results for changes in initial concentration (1.1, 6.8, 21.5 mg L^−1^). pH initially set to 5.5, temperature 22 ± 2 °C, contact time 4 hours. Error bars represent the standard deviation (*n* = 2, samples were repeated if differing by ±5% from mean)

Sorbent	Result	Initial concentration (mg Cd per L)		
		1.1	6.8	21.5
Grape wastes	Removal efficiency (%)	95.9 ± 1.24	96.0 ± 0.51	92.8 ± 0.77
	Capacity (mg g^−1^)	0.22 ± 0.003	1.30 ± 0.007	3.99 ± 0.033
	Equilibria conc. (mg L^−1^)	0.046 ± 0.01	0.27 ± 0.03	1.55 ± 0.16
	Final pH	7.67	7.15	6.49
Flax wool	Removal efficiency (%)	90.4 ± 0.43	89.4 ± 0.31	78.1 ± 0.05
	Capacity (mg g^−1^)	0.20 ± 0.001	1.21 ± 0.004	3.36 ± 0.002
	Equilibria conc. (mg L^−1^)	0.11 ± 0.005	0.72 ± 0.021	4.70 ± 0.01
	Final pH	6.97	6.64	6.36

The maximum adsorption capacities found in this study are 3.99 and 3.36 mg Cd per g for grape waste and flax wool respectively. At much higher initial concentrations this value would be higher, albeit at unrealistic conditions, *i.e.* not found in environmental/waste effluents. The uptake is compared with the performance of other (modified) waste materials in Table 3 showing that the studied unmodified AWBs offer competitive removal compared with modified waste. Some modified AWB materials in the literature reported to have been activated, carbonised or ground appear to have larger maximum Cd uptake capacities than unmodified AWBs (Table 3) on a per gram basis. For example, a composite involving CaCO_3_ nanoparticles deposited onto porous sewage sludge biochar, which is a more energy intensive and costly material than AWBs, achieved about 10 times greater removal capacity.^35^

**Table 3:** Comparison of maximum adsorption capacities (mg of Cd per g dry sorbent) of grape wastes and flax wool with the performance of other AWB materials and activated carbons reported in the literature

Sorbent material	Capacity – at maximal condition of adsorption (mg Cd per g sorbent)	Ref.
**Grape wastes**	**3.99**	**This study**
**Flax wool**	**3.36**	**This study**
Grape stalks	27.9	27
Corn stalk	3.81	37
Modified wheat straw	39.22	38
Rice husk ash	3.04	39
Microwaved olive stone activated carbon	11.72	40
Ground sugarcane bagasse	69.06	41
Ground maize corncob	105.6	41

Further study is required however into the overall cost of pre-treatments and modifications to determine if the improved capacity and performance they provide are worth the costs, complexity and chemicals added to the process. Maximum adsorption capacity, although being highly valued for adsorbent materials, may not be as important for AWBs materials since their low cost or presence as by-products otherwise requiring disposal mean that larger masses of sorbent may be applied in the adsorption process, offsetting their lower adsorption capacities. Conversely, this means that higher post-treatment disposal volumes of adsorbent are generated, although the final disposal route will depend on local regulatory classifications (*i.e.* classification of the material as a waste or usable biomass) and thresholds, and the potential of the AWBs for further processing and valorisation.^36^

### 3.4 Sorption kinetics

**Fig. 4:**
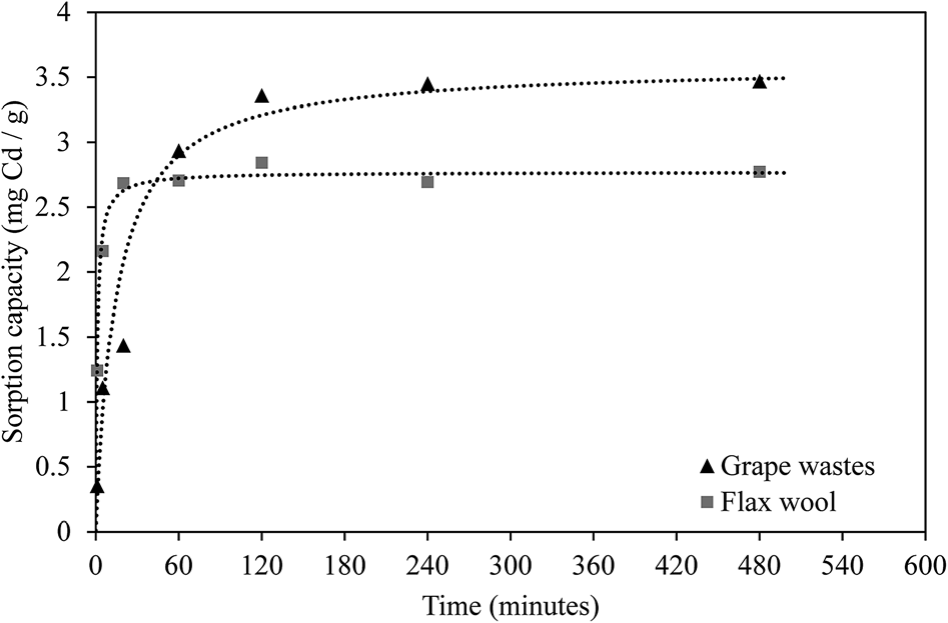
Cadmium sorption kinetics onto grape wastes (GW, black triangles) and flax wool (FW, grey squares) as a function of time for an initial concentration of ∼18.4 mg L^−1^, pH of 5.5 and temperature of 22 °C ± 2 °C (*n* = 2). Lines represent pseudo second-order modelled data.

Kinetic experiments were undertaken to establish the period taken by each adsorbent to reach equilibrium. The results are presented in Fig. 4 and show that Cd adsorption occurred very rapidly onto FW whereas sorption occurred more slowly onto GW (8 and 183 min according to the pseudo-second order model (*r*^2^ > 0.998), respectively).

The experimental data were fitted using different models (Table 4) to elucidate mechanisms controlling the uptake of Cd by these AWBs. The sorption of Cd to grape wastes and flax wool fits very well to a pseudo second-order model. This indicates that the kinetic rate is partly influenced by chemisorption mechanisms^42^ and is partly complex in nature as it does not fit as well to a pseudo first-order model. Other studies using AWB materials as sorbents have also found kinetic experimental data to fit this model, possibly owing to chemisorption due to the interaction of the contaminants with the functional groups in cellulose and hemicellulose material.^43^ Interestingly, the uptake here was not related to the surface area, whereas in chemisorption uptake would generally be correlated with this parameter. This lack of correlation was observed in other sorption processes elsewhere,^44^ and it was attributed to the existence of specific sites within the sorbent with affinity for the contaminant.

**Table 4:** Pseudo first- and pseudo second-order kinetic modelled data – quantity sorbed at equilibrium (*Q*_e_, mg Cd per g sorbent), rate constants and *R*^2^ values

	Pseudo first-order			Pseudo second-order		
	*Q*_e_ (mg Cd per g)	Rate constant (min^−1^)	*R* ^2^ value	*Q*_e_ (mg Cd per g)	Rate constant (mg (g min)^−1^)	*R* ^2^ value
GW	2.73	0.0223	0.9746	3.59	0.0196	0.9981
FW	1.23	0.0435	0.6582	2.77	0.340	0.9997

The sorption kinetics rate associated with flax wool (0.340 mg (g minute)^−1^) was greater than the equivalent rate for grape wastes (0.0196 mg (g minute)^−1^). This lower rate for grape wastes may indicate that chemisorption processes such as ion-exchange dominate in grape waste whereas van der Waals forces may dominate in the uptake of Cd in flax wool.^45^ These results point out the important potential of flax wool for its use in the filtration of contaminated effluents, or either flax wool or grape waste (in a suitable form) for the passive treatment of diffuse contamination such as leachates in mine sites where other more expensive technologies like dispersed alkaline substrate are currently implemented.^46^

### 3.5 Effect of surface chemistry on the uptake of Cd by grape waste

**Fig. 5:**
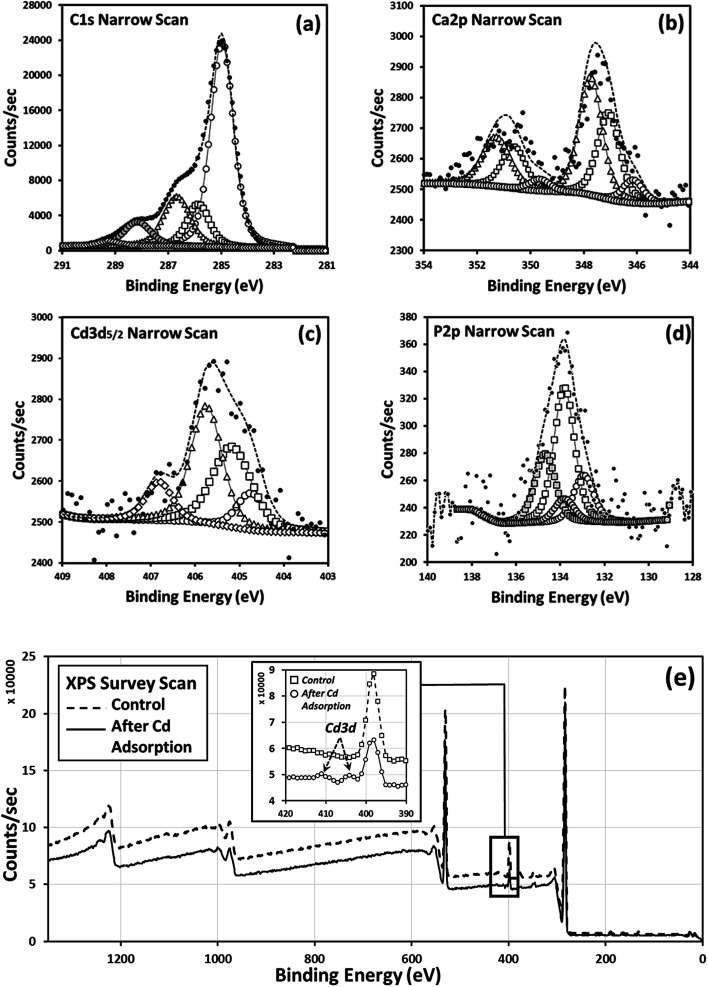
XPS spectra from grape waste showing available surface functional groups in dried grape peel: (a) C 1s narrow scan peak de-convolution, (b) Ca narrow scan peak de-convolution, (c) narrow scan peak de-convolution showing interaction of Cd with functional groups in grapes incubated with Cd (0.2 g grape waste incubated with 40 mL of 20 mg Cd per L for 48 h) (d) P 2p narrow scan peak de-convolution and (e) comparison survey spectra of control and Cd adsorbed grape waste, inset, zoomed in area showing Cd 3d peak and nitrogen N 1s peaks. The narrow scan peak deconvolution data (a–d) illustrating the interaction of Cd with grape waste is also given.

The surface chemistry of grape waste, following incubation with Cd (*i.e.* following batch adsorption studies), was investigated for the first time *via* XPS as detailed in Tables. 5–9. Through XPS, chemisorption of Cd to specific functional groups of the waste material (leading to stable capture of the contaminant) can be identified. It was not possible to reliably analyse the flax wool *via* XPS due to its fibrous nature, which made sample immobilisation (without chemical pre-treatment) impractical. The survey scan for grape waste (Fig. 5(e)) showed the existence of possible elements that could chemically interact with Cd. Further investigation of the carbon chemistry and the Ca content is shown in the deconvolution of the peak data (Fig. 5(a) and (b)), specifically it can be interpreted that Cd can interact with carbonate, sulphur, and to a lesser extent oxygen (which could be from polyphenols, in agreement with complexation found in flax^33^) groups (Fig. 5(c)) present in the dry waste. Part of this interaction could have resulted from the displacement of calcium originally in these sites through ion exchange, as has been proposed in previous work carried out on other types of sorbents.^35^

**Table 5:** XPS spectra from grape waste showing available surface functional groups in dried grape peel: C 1s narrow scan peak de-convolution

Peak assignments	C–C, C–H (sp^3^)	Amine (C–N)	C–O alcohol/ether	N–C=O amide	Carbonate (–CO_3_)
Peak BE	∼285.0 eV	∼285.9 eV	∼286.7 eV	∼288.2 eV	∼289.2 eV
Atomic %	60.53	12.36	17.33	8.49	8.8 ± 0.21

**Table 6:** XPS spectra from grape waste showing available surface functional groups in dried grape peel: Ca narrow scan peak de-convolution

Peak assignments	CaO	CaCO_3_	Ca_3_(PO_4_)_2_
Peak BE	∼346.2 eV	∼347.1 eV	∼347.7 eV
Atomic %	13.24	36.3	54.46

**Table 7:** XPS spectra from grape waste showing available surface functional groups in dried grape peel: narrow scan peak de-convolution showing interaction of Cd with functional groups in grapes incubated with Cd (0.2 g grape waste incubated with 40 mL of 20 mg Cd per L for 48 h)

Peak assignments	Cd	CdCO_3_	CdS (or Cd^2+^)	CdO
Peak BE	∼404.75eV	∼405.18 eV	∼405.74 eV	∼406.81 eV
Atomic %	18.92	32.67	32.96	10.45

**Table 8:** XPS spectra from grape waste showing available surface functional groups in dried grape peel: P 2p narrow scan peak de-convolution

Peak assignments	Ca_3_(PO_4_)_2_	CaHPO_4_
Peak BE	132.9 eV	133.8 eV
Atomic %	25.7	74.3

XPS spectra from grape waste showing available surface functional groups in dried grape peel: comparison survey spectra of control and Cd adsorbed grape waste, inset, zoomed in area showing Cd 3d peak and nitrogen N 1s peaks

**Table:** 

Peak assignments	O 1s	N 1s	Ca 2p	C 1s	P 2p	Cd 3d
Grape waste after Cd adsorption						
Peak BE (eV)	530	399	347	285	132	403
Atomic %	18.7	3.1	0.4	77.3	0.30	0.20

**Table:** 

Peak assignments	O 1s	N 1s	Ca 2p	C 1s	P 2p	Cd 3d	S 2p	K 2s
Control dry grape waste								
Peak BE (eV)	530	399	347	285	132	403	163	375
Atomic %	19.9	5.9	0.4	72.4	0.5	0.0	0.2	0.8

## 4 Concluding remarks

The efficiency of various globally abundant AWBs for the adsorption of Cd has been investigated for the first time. Cd is a priority pollutant that causes damage to multiple organs and can leach to freshwater in effluents from a wide range of industries, landfills and mines. The clean-up properties of the AWBs have been studied under conditions relevant to effluents contaminated with Cd, such as mine run off, which is one of the environmental problems where they could be used. Grape wastes and flax wool are very effective in the removal of Cd from aqueous solution, the former removing >90% of Cd from initial concentrations from 1.1–21.5 mg Cd per L and outperformed the more expensive GAC used in tertiary treatment (which only removed 34% of the Cd from the lower initial concentration tested). Flax wool has exceptionally fast kinetics (equilibria was reached in 8 min when the concentration of Cd was 18.4 mg L^−1^), and as the rate of sorption is very important in adsorption systems, flax wool shows a higher potential if used practically in filtration systems. The mechanism of removal of Cd using grape waste relates to the chemical interaction of Cd with oxygen (possibly from polyphenols), sulphur, and carbonate groups, and possible displacement of Ca from the original sorbents.

This study has demonstrated the potential of low cost and low/un-processed sorbents for effective removal of Cd: flax wool and grape waste but also flax shive, wheat and oat straw. Further work should address optimising their final form for their application in environmental remediation (*e.g.* as mulches on soil alongside water courses, or as baled (*i.e.* bundled and bound) material in contaminated waters). AWBs are often available in large quantities, and so can be useful at large scale as they require minimal technical provision/input and reduce the need for chemical additions or costlier processes. While further work is required on their application to other divalent metal cations, this research indicates that some AWBs can be directly (*i.e.* without pre-treatment or modification) used in bulk to remediate effluents contaminated with heavy metals, without requiring further cost or energy input, making them potentially suitable for low-cost treatment of persistent (*e.g. via* mine drainage) or acute (*e.g.* spillages) discharges in rural and other areas.

## Conflicts of interest

There are no conflicts to declare.

## Supplementary Material
